# Supplementary material for: Mechanisms governing the pioneering and redistribution capabilities of the non-classical pioneer PU.1
Source: Nat Commun. 2020 Jan 21;11:402. doi: 10.1038/s41467-019-13960-2 (PMC6972792; doi:10.1038/s41467-019-13960-2)
Supplement: Supplementary file 7 — Source data [file 41467_2019_13960_MOESM7_ESM.zip › Source_Data/FigureS2/FigureS2B_MotifScanOutput/KmeansCluster5/homerResults/motif4.similar.html]

motif4

## Information for motif4

A
T
G
C
A
C
G
T
C
G
A
T
A
G
T
C
A
C
G
T
C
A
G
T
A
G
T
C
A
C
G
T
  
Reverse Opposite:  

G
T
C
A
A
C
T
G
G
T
C
A
T
G
C
A
A
C
T
G
G
C
T
A
G
T
C
A
A
T
C
G
  

|  |  |
| --- | --- |
| p-value: | 1e-263 |
| log p-value: | -6.059e+02 |
| Information Content per bp: | 1.721 |
| Number of Target Sequences with motif | 1796.0 |
| Percentage of Target Sequences with motif | 45.13% |
| Number of Background Sequences with motif | 8344.8 |
| Percentage of Background Sequences with motif | 19.49% |
| Average Position of motif in Targets | 97.0 +/- 54.8bp |
| Average Position of motif in Background | 100.1 +/- 76.9bp |
| Strand Bias (log2 ratio + to - strand density) | 0.1 |
| Multiplicity (# of sites on avg that occur together) | 1.10 |
| Motif File: | file (matrix) reverse opposite |

### Similar de novo motifs found

|  |  |  |  |  |  |  |  |
| --- | --- | --- | --- | --- | --- | --- | --- |
| Rank | Match Score | Redundant Motif | P-value | log P-value | % of Targets | % of Background | Motif file |
| 1 | 0.821 | A C T G T C G A C G T A A C T G C G T A C T G A A C T G | 1e-125 | -287.923291 | 46.73% | 28.02% | motif file (matrix) |
| 2 | 0.817 | A G T C G A C T C G T A A T G C G A C T A G C T G A T C C G A T A C T G G A T C G C A T | 1e-67 | -154.476468 | 25.13% | 14.12% | motif file (matrix) |
| 3 | 0.806 | C T G A A G T C A C G T A C G T A G T C A C G T A C T G A G T C C G A T | 1e-65 | -150.947280 | 6.51% | 1.62% | motif file (matrix) |
| 4 | 0.924 | T G C A A T C G T A G C T C G A A T C G T C G A G T C A A T C G | 1e-49 | -114.087841 | 10.48% | 4.45% | motif file (matrix) |
